# Alterations in the Physicochemical and Structural Properties of a Ceramic–Polymer Composite Induced by the Substitution of Hydroxyapatite with Fluorapatite

**DOI:** 10.3390/ma18194538

**Published:** 2025-09-29

**Authors:** Leszek Borkowski, Krzysztof Palka, Lukasz Pajchel

**Affiliations:** 1 Chair and Department of Biochemistry and Biotechnology, Medical University of Lublin, Chodzki 1, 20-093 Lublin, Poland; 2 Faculty of Mechanical Engineering, Lublin University of Technology, Nadbystrzycka 36, 20-618 Lublin, Poland; k.palka@pollub.pl; 3 Chair of Analytical Chemistry and Biomaterials, Department of Analytical Chemistry, Medical University of Warsaw, ul. Banacha 1, 02-097 Warsaw, Poland

**Keywords:** biomaterials’ characterization, ceramic–polymer composite, hydroxyapatite (HAP), fluorapatite (FAP), scaffold microarchitecture, surface topography

## Abstract

In recent years, apatite-based materials have garnered significant interest, particularly for applications in tissue engineering. Apatite is most commonly employed as a coating for metallic implants, as a component in composite materials, and as scaffolds for bone and dental tissue regeneration. Among its various forms, hydroxyapatite (HAP) is the most widely used, owing to its natural occurrence in human and animal hard tissues. An emerging area of research involves the use of fluoride-substituted apatite, particularly fluorapatite (FAP), which can serve as a direct fluoride source at the implant site, potentially offering several biological and therapeutic advantages. However, substituting HAP with FAP may lead to unforeseen changes in material behavior due to the differing physicochemical properties of these two calcium phosphate phases. This study investigates the effects of replacing hydroxyapatite with fluorapatite in ceramic–polymer composite materials incorporating β-1,3-glucan as a bioactive polymeric binder. The β-1,3-glucan polysaccharide was selected for its proven biocompatibility, biodegradability, and ability to form stable hydrogels that promote cellular interactions. Nitrogen adsorption analysis revealed that FAP/glucan composites had a significantly lower specific surface area (0.5 m^2^/g) and total pore volume (0.002 cm^3^/g) compared to HAP/glucan composites (14.15 m^2^/g and 0.03 cm^3^/g, respectively), indicating enhanced ceramic–polymer interactions in fluoride-containing systems. Optical profilometry measurements showed statistically significant differences in profile parameters (e.g., Rp: 134 μm for HAP/glucan vs. 352 μm for FAP/glucan), although average roughness (Ra) remained similar (34.1 vs. 27.6 μm, respectively). Microscopic evaluation showed that FAP/glucan composites had smaller particle sizes (1 μm) than their HAP counterparts (2 μm), despite larger primary crystal sizes in FAP, as confirmed by TEM. XRD analysis indicated structural differences between the apatites, with FAP exhibiting a reduced unit cell volume (524.6 Å^3^) compared to HAP (528.2 Å^3^), due to substitution of hydroxyl groups with fluoride ions. Spectroscopic analyses (FTIR, Raman, ^31^P NMR) confirmed chemical shifts associated with fluorine incorporation and revealed distinct ceramic–polymer interfacial behaviors, including an upfield shift of PO_4_^3−^ bands (964 cm^−1^ in FAP vs. 961 cm^−1^ in HAP) and OH vibration shifts (3537 cm^−1^ in FAP vs. 3573 cm^−1^ in HAP). The glucan polymer showed different hydrogen bonding patterns when combined with FAP versus HAP, as evidenced by shifts in polymer-specific bands at 888 cm^−1^ and 1157 cm^−1^, demonstrating that fluoride substitution significantly influences ceramic–polymer interactions in these bioactive composite systems.

## 1. Introduction

Fluorapatite (FAP) (Ca_10_(PO_4_)_6_F_2_) is a mineral with growing significance across multiple industries. According to a report by Global Market Insights, the fluorapatite market is projected to expand considerably from 2023 to 2032, driven by its widespread application in the production of phosphoric acid and hydrogen fluoride. In the pharmaceutical sector, hydrogen fluoride is a precursor for hydrofluoric acid, which serves as a key starting material in the synthesis of fluorinated pharmaceutical compounds [1]. In the electromechanical industry, synthetic FAP doped with manganese (II) and antimony (V) formed the basis of second-generation fluorescent tube phosphors [2,3,4].

Because calcium phosphates, particularly fluorine-substituted variants, are naturally present in the human body, FAP has gained increasing interest in biomedical applications. Nanoscale FAP is particularly relevant in conservative dentistry and dental surgery, where it is used in toothpaste formulations, dental cements, and implant coatings [5,6,7,8,9,10]. It is also applied in orthopedics, both as a coating for bone implants [11,12] and as a component in bone substitute materials [13]. Given the expanding body of research on fluorapatite, it is evident that FAP plays a vital role in both industrial and medical contexts, with promising implications for human and veterinary medicine.

FAP shares a similar chemical formula and many properties with hydroxyapatite (HAP) (Ca_10_(PO_4_)_6_(OH)_2_), the most commonly used apatite ceramic in biomedicine. Both exhibit typical bioceramic characteristics such as porosity, brittleness, high compressive strength, bioactivity, and non-toxicity [14,15]. However, FAP offers notable advantages over HAP, including greater chemical stability, lower solubility, and slower dissolution rates [16,17,18]. In our previous work, we demonstrated that FAP has a higher density and lower porosity than HAP, regardless of sintering conditions [19,20].

Like other apatites, FAP can be combined with organic polymers to form ceramic–polymer composites. Despite their potential, FAP-based composites remain underexplored. These composites typically integrate the mechanical strength, biocompatibility, and bioactivity of the ceramic phase with the flexibility and processability of the polymer phase, resulting in materials with improved performance for medical applications [21,22,23,24,25,26,27,28,29,30]. One such example is the fluorapatite/1,3-β-glucan (FAP/glucan) composite, which we previously developed and characterized, demonstrating promising biological activity in in vitro cell culture experiments [31].

A fundamental aspect of regenerative medicine is understanding how biomaterials interact with cells. It is well established that the mechanical properties, surface chemistry, and topography of biomaterials significantly influence cellular responses [32,33]. This study aims to provide a comprehensive comparative analysis of the physicochemical properties of ceramic–polymer scaffolds in which HAP is partially or fully substituted with FAP. Understanding how this substitution alters material properties is critical, as even subtle changes may affect biological performance and cellular behavior.

To achieve this, two types of ceramic–polymer composites—FAP/glucan and HAP/glucan—were compared. A wide array of analytical techniques was employed to characterize their structural and surface properties, including optical profilometry, micro-computed tomography (microCT), transmission electron microscopy (TEM), and gas adsorption porosimetry. Additionally, spectroscopic and diffraction techniques—including X-ray diffraction (XRD), Raman spectroscopy, Fourier transform infrared (FTIR) spectroscopy, and nuclear magnetic resonance (NMR)—were employed to provide a comprehensive understanding of the compositional and structural changes resulting from the substitution of HAP with FAP.

This work provides comparative analysis of the effect of hydroxyapatite substitution with fluorapatite on the structure and surface properties of ceramic–polymer composites, which is crucial for their future application in regenerative medicine. The use of multispectral analytical techniques enables a thorough assessment of changes in the material’s microstructure and chemistry, representing a novel approach to the optimization of apatite-based biomaterials which may contribute to the development of more stable, bioactive biomaterials. Solving this problem is of significant global importance in the context of the growing demand for durable and safe materials for bone and dental tissue regeneration.

## 2. Materials and Methods

### 2.1. Composite Sample Preparation

The first stage involved the synthesis of fluorapatite (FAP) and hydroxyapatite (HAP) granules, following the procedure outlined in Polish patent no. PL 235803 [34] and previous studies [19,20]. The apatite powders were sintered at 800 °C for 2 h, yielding granules with diameters ranging from 0.2 to 0.3 mm. These granules were then combined with a polysaccharide polymer (Curdlan, Wako Chemicals, Osaka, Japan) according to the method outlined in Polish patent no. 236369 [35]. The ceramic/glucan mixture, composed of 83 wt.% granules and 17 wt.% β-1,3-glucan powder, was placed into a glass mold and heated at 95 °C for 15 min to induce gelation. The gelled composites were then sectioned into flat discs approximately 10 mm in diameter and 2 mm thick using a scalpel. Finally, the samples were sealed in plastic/paper peel pouches and sterilized by exposure to ethylene oxide at 55 °C for 1 h.

The materials obtained were named as follows:

HAP: hydroxyapatite granules;
FAP: fluorapatite granules;
HAP/glucan: HAP-granules-based composites, containing β-1,3-glucan as a binder;
FAP/glucan: FAP-granules-based composites, containing β-1,3-glucan as a binder.

### 2.2. Grinding and Sample Preparation for Imaging and Analyses

Prior to the microscopic imaging, TEM, PXRD, Raman, FTIR, and NMR measurements, the samples were pulverized and homogenized by grinding them in a cryogenic grinder (6770 Freezer/Mill, SPEX SamplePrep LCC, Metuchen, NJ, USA). The process involved two steps repeated ten times: freezing for 1 min and grinding for 1 min repeated 10 times.

### 2.3. Chemical and Phase Composition Analysis

#### 2.3.1. X-Ray Diffractometry (XRD)

Powder X-ray diffraction (PXRD) of the HAP/glucan and FAP/glucan composite samples was carried out using a Bruker D8 Advance diffractometer (Bruker, Karlsruhe, Germany). The measurements were performed with CuKα radiation (λ = 1.54 Å) over a 2θ range of 10° to 70°, with a step size of 0.03°. To estimate crystallite size, the full width at half maximum (FWHM) was determined for the (002) and (300) diffraction peaks, corresponding to the crystallite dimensions along the c-axis and a-axis, respectively. Crystallite sizes were calculated using the Scherrer equation [36].

#### 2.3.2. Characterization by Raman Spectroscopy

Raman spectra were acquired using an iRaman 532 spectrometer (B&W Tek, Newark, DE, USA) under the following conditions: 20× objective lens magnification, 5000 accumulations with an integration time of 1000 ms each, and 100% laser power. The excitation wavelength was 532 nm. Spectral data were collected in the range of 170–4000 cm^−1^ at room temperature.

#### 2.3.3. Fourier Transform Infrared Spectroscopy (FTIR) Measurements

FTIR measurements were conducted using a Spectrum 1000 spectrometer (Perkin Elmer, Cleveland, OH, USA) operating in the mid-infrared range. Spectra were acquired at a resolution of 2 cm^−1^ with 50 scans per sample. Each sample was mixed with potassium bromide (KBr) in a 1:100 weight ratio and pressed into pellets prior to measurement. The spectral range recorded was 400–4000 cm^−1^.

Raman and FTIR spectra were analyzed using GRAMS/AI 8.0 software (Thermo Scientific, Burlington, ON, USA, 2006). Data processing included baseline correction, second-derivative transformation, and peak fitting.

#### 2.3.4. Nuclear Magnetic Resonance Spectroscopy (NMR)

The NMR spectra of ^31^P, ^19^F, ^13^C, and 1H nuclei were recorded under magic angel spinning MAS using a 700 MHz Agilent DirectDrive2 spectrometer (Agilent Technologies, Santa Clara, CA, USA)). For the ^31^P, ^19^F, ^13^C, and ^1^H MAS NMR experiments, the samples were spun at 10 kHz (^31^P, ^13^C) and 20 kHz (^19^F, ^1^H). In the ^31^P and C experiments, conventional single pulse-acquire (Bloch decay, BD) and cross-polarization (CP, ^1^H→^31^P, ^19^F→^31^P, ^1^H→^13^C) techniques were used. In the case of the ^1^H and ^19^F MAS NMR, only single pulse-acquire (Bloch decay) spectra were acquired. For all experiments, 32 scans were acquired. The peak fittings were performed using the NutsPro (Acorn NMR, Livermore, CA, USA, USA, 2007) computer programs.

### 2.4. Structural and Microarchitectural Analysis

#### 2.4.1. Optical Microscopy

A Keyence VHX-7000 ultra-high-precision digital microscope (Keyence International, Mechelen, Belgium) was used to examine the morphology of the ceramic and β-1,3-glucan composites following cryogenic milling. The microscope’s integrated image analysis system allows for both 2D and 3D visualization, as well as accurate measurement of various microparticle dimensions.

#### 2.4.2. Electron Microscopy and Elemental Analysis

The morphology of the samples was analyzed using a transmission electron microscope (JEM 1400, JEOL, Tokyo, Japan) equipped with a full-range energy-dispersive X-ray spectroscopy (EDS) system (INCA Energy TEM, Oxford Instruments, Abingdon, UK). A droplet of the sample suspended in ethanol was deposited onto a copper grid coated with a formvar film, left to dry, and then examined at an accelerating voltage of 80 kV.

#### 2.4.3. MicroCT-Based Analysis of Internal Microarchitecture

Before evaluation samples were dried for 72 h at 37 °C, the microstructure of the composite samples was assessed via micro-computed tomography (microCT; Xradia 510 Versa, Carl Zeiss, Dublin, CA, USA) with a voxel size of 4.85 µm. Cross-sectional images were reconstructed using Reconstructor software (version 16.1.13038, Carl Zeiss).

### 2.5. Surface and Porosity Characterization

#### 2.5.1. Determination of Specific Surface Area and Micropore Distribution by Low-Temperature Nitrogen Adsorption

Porosity measurements were carried out on three samples of each composite using a sorption analyzer (ASAP 2020C, Micromeritics, Norcross, GA, USA). Prior to analysis, all samples were degassed at 60 °C. The specific surface area (SBET) was calculated using the Brunauer–Emmett–Teller (BET) multilayer adsorption theory. The average mesopore diameter was estimated using the equation Dp = 4Vp/SBET. Additional parameters determined included micropore surface area (Smicro), external surface area (Sext), total pore volume, and median pore width. Statistical significance was assessed using an unpaired *t*-test, with differences considered significant at *p* < 0.05 (GraphPad Prism 7.04, San Diego, CA, USA).

#### 2.5.2. Surface Topographical Analysis Using 3D Optical Profilometer

Three-dimensional surface profiles of the samples were analyzed using a Bruker Contour GT-I 3D optical profilometer. (Bruker, Tucson, AZ, USA). The measured roughness parameters included: average surface roughness (Ra), maximum peak height (Rp), root mean square roughness (Rq), total profile height (Rt), and maximum valley depth (Rv). A comprehensive description of these surface roughness parameters is available in Supplementary Figure S1. For each sample, ten measurements were performed. Statistical analysis was conducted using the Student’s unpaired *t*-test, with significance determined via GraphPad Prism 7.04 software.

## 3. Results and Discussion

Ceramic–polymer composites represent a promising approach to biomaterial design, combining the bioactivity of calcium phosphates with the flexibility and processability of polymeric matrices. The selection of β-1,3-glucan as the polymeric component was based on its unique gelation properties and proven biocompatibility in tissue engineering applications. This polysaccharide forms stable hydrogels that create intimate interfacial contact with ceramic particles while maintaining structural integrity under physiological conditions.

The fundamental premise of this research is that substituting hydroxyapatite with fluorapatite in glucan-based composites will result in measurable changes in physicochemical properties due to inherent structural differences between these calcium phosphate phases. The ionic size difference between hydroxyl and fluoride ions leads to distinct crystal lattice parameters, surface chemistry, and interfacial behavior with the polymer matrix.

In glucan-based composites, the polysaccharide chains form hydrogen bonds with surface groups on apatite crystals, creating a network structure that influences porosity, surface area, and particle distribution. The replacement of surface hydroxyl groups with fluoride ions in fluorapatite is expected to alter these interfacial interactions, potentially modifying the ceramic–polymer binding characteristics and resulting composite properties.

This systematic comparative analysis aims to quantify how fluoride substitution in the ceramic phase influences the structural, chemical, and surface properties of β-1,3-glucan composites. Understanding these structure–property relationships is essential for optimizing composite formulations for biomedical applications where controlled interfacial stability is a critical performance parameter.

### 3.1. Analysis of the Phase Composition

**Figure 1:**
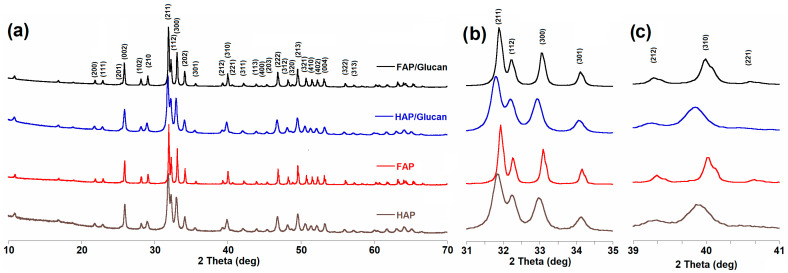
PXRD diffractograms of HAP, FAP, HAP/glucan, and FAP/glucan samples: (**a**) 2Θ range from 20 to 70°; (**b**) 2Θ range from 31 to 35° (magnified ×5); and (**c**) 2Θ range from 39 to 41.0° (magnified ×5).

Figure 1a presents the PXRD patterns for all FAP and HAP granules, as well as for composites based on FAP and HAP granules incorporating β-1,3-glucan as a binder. The diffractograms display the main characteristic reflections of fluoridated apatites and hydroxyapatite, which correspond well to JCPDS: 09-432 and JCPDS: 15-876, respectively. Rietveld refinement confirmed the presence of a pure apatite phase in the HAP and HAP/glucan samples, and a pure fluorapatite phase in the FAP and FAP/glucan samples.

The diffractograms reveal that all expected reflections from hydroxyapatites and fluoridated apatites were present and were conspicuous and well separated. A notable characteristic observed in the HAP/glucan and FAP/glucan samples was the presence of broader peaks compared to HAP and FAP granules. This peak broadening is commonly observed in composite materials due to the presence of the polymeric phase and potential strain effects during composite formation, as well as the amorphous nature of the glucan binder which can introduce structural disorder at the ceramic–polymer interface [37].

As documented in previous studies of hydroxyapatite and fluorapatite samples, the diffractogram peaks (2 1 1), (3 0 0) and (2 0 2) according to 2θ values at 31.7°, 32.8°, and 34.0° are systematically shifted to higher angles for fluorapatite compared to hydroxyapatite (Figure 1b). These shifts to higher 2θ values were caused by a decrease in the a-axis length of the hexagonal crystal lattice, induced by the substitution of larger hydroxyl ions (OH^−^, ionic radius 140 pm) with smaller fluoride ions (F^−^, ionic radius 133 pm) [20,38]. A similar shift was observed for peak (3 1 0) around 40.0 degrees (Figure 1c), confirming the successful incorporation of fluoride ions into the apatite crystal structure. This systematic shift in reflection positions is a definitive indicator of fluoride substitution and has been consistently reported in the literature as a key characteristic of fluorinated apatites.

The quantitative analysis through Rietveld refinement revealed significant differences in lattice parameters between the materials (Table 1). The calculated cell parameters showed that the lattice parameter was consistently smaller for FAP samples (9.379(3) Å) compared to HAP samples (9.415(2) Å). Similarly, the calculated cell volume decreases from 528.2(1) Å^3^ for HAP to 524.6(3) Å^3^ for FAP samples, representing a volume reduction of approximately 0.7%. This cell volume contraction is directly attributable to the ionic size difference between OH^−^ and F^−^ ions and results in a denser crystal structure [20]. The same relationships were observed in the diffractograms obtained for HAP/glucan and FAP/glucan composites, with cell volumes of 528.7(3) Å^3^ and 525.0(3) Å^3^, respectively, indicating that the composite formation process does not significantly alter the fundamental crystal structure of the ceramic phase.

Crystallite size analysis using the Scherrer equation revealed interesting differences between the materials. FAP samples consistently showed larger crystallite sizes along both the c-axis (58 ± 4 nm) and a-axis (49 ± 4 nm) compared to HAP samples (30 ± 2 nm along the c-axis and 24 ± 2 nm along the a-axis). This increase in crystallite size in fluorinated samples can be attributed to the enhanced thermal stability and crystallinity induced by fluoride incorporation. The fluoride substitution has been shown to stabilize the apatite structure against thermal decomposition and promote crystal growth during the thermal treatment process. The composite samples maintained similar crystallite sizes to their corresponding pure ceramic phases, with FAP/glucan showing values of 51 ± 3 nm (c-axis) and 47 ± 3 nm (a-axis), and HAP/glucan showing 34 ± 2 nm (c-axis) and 31 ± 2 nm (a-axis). The slight reduction in crystallite size in the composite materials compared to the pure ceramics may be attributed to the constraining effect of the polymeric matrix on crystal growth during thermal processing.

The structural analysis provides insights into the degree of fluoride substitution in the synthesized fluorapatite. Based on the lattice parameter values obtained, the materials synthesized in this study exhibit characteristics of fluorohydroxyapatite (FOHAP) rather than pure fluorapatite. The intermediate values of lattice parameters between those reported for pure hydroxyapatite (a = 9.421 Å, c = 6.882 Å; JCPDS: 09-432) and pure fluorapatite (a = 9.367 Å, c = 6.884 Å; JCPDS: 15-876) suggest partial fluoride substitution. This incomplete substitution is commonly observed in sol–gel synthesis methods and is consistent with spectroscopic evidence that will be discussed in subsequent sections. The partial substitution does not significantly compromise the beneficial properties of fluoride-containing apatites and may actually offer advantages in terms of bioactivity and controlled fluoride release in biological applications.

The comparison between granules and composites reveals that the incorporation of β-1,3-glucan as a polymeric binder does not induce significant structural changes in the ceramic phase. This is evidenced by the preservation of characteristic apatite reflections and similar lattice parameters in both pure ceramic and composite materials. The slight peak broadening observed in composite samples is attributed to the presence of the amorphous polymeric phase and potential microstrain effects at the ceramic–polymer interface, but does not indicate any detrimental phase transformations or chemical interactions between the ceramic and polymer phases. This structural stability is crucial for maintaining the bioactive properties of the ceramic component while benefiting from the mechanical advantages provided by the polymer matrix.

The results obtained in this study are consistent with the broader literature on fluoride-substituted apatites. The systematic shift in diffraction peaks to higher angles, the reduction in unit cell volume, and the enhanced crystallinity are all well-documented characteristics of fluorinated apatites. The maintenance of phase purity at relatively low sintering temperatures (800 °C) is particularly advantageous for biomedical applications, as it avoids the formation of less biocompatible secondary phases while preserving the beneficial properties of both the ceramic and polymer components.

In conclusion, the XRD analysis confirms the successful synthesis of phase-pure fluoride-substituted apatite and hydroxyapatite composites with well-defined structural characteristics. The fluoride substitution results in predictable structural modifications without compromising phase purity, and the incorporation of the glucan binder does not adversely affect the crystal structure of the ceramic phases. These findings provide a solid foundation for understanding the structure–property relationships in these bioactive composite materials and support their potential application in bone tissue engineering applications.

### 3.2. Raman Spectroscopy

**Figure 2:**
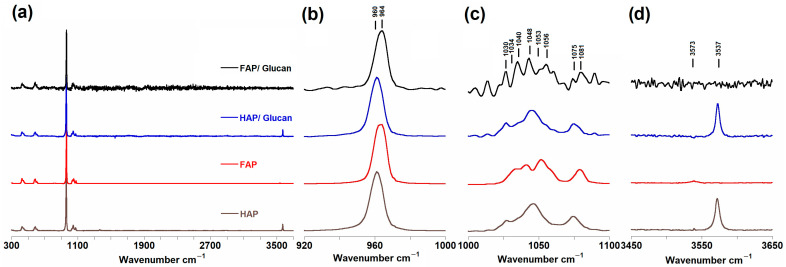
Raman spectra of HAP, FAP, HAP/glucan, and FAP/glucan samples: (**a**) range 300 to 3700 cm^−1^; (**b**) range 920 to 1000 cm^−1^; (**c**) range 1000 to 1100 cm^−1^ (magnified ×10); and (**d**) range 3450 to 3650 cm^−1^ (magnified ×5).

The Raman spectra obtained for all investigated samples are presented in Figure 2a. For comparative analysis, all spectra were normalized to the most intense ν_1_ PO_4_^3−^ band, which appeared at 961 cm^−1^ for the unsubstituted composites and at 964 cm^−1^ for the fluoride-substituted samples. The Raman spectroscopic analysis revealed characteristic vibrational bands typical for both HAP and FAP phases. All obtained spectra exhibited the dominant PO_4_^3−^ band around 960 cm^−1^, accompanied by two distinct spectral regions containing multiple bands at 1000–1100 cm^−1^ and 400–650 cm^−1^. Additionally, both HAP and FAP samples displayed a characteristic weak band in the 3500–3600 cm^−1^ region, attributed to structural OH group vibrations.

The most significant spectroscopic observation was the upfield shift of the ν_1_ PO_4_^3−^ band from 961 cm^−1^ in HAP granules and HAP/glucan composites to 964 cm^−1^ in FAP granules and FAP/glucan samples (Figure 2b). This 3 cm^−1^ shift toward higher wavenumbers provides direct evidence of P-O bond shortening within the phosphate tetrahedra upon fluoride incorporation. The mechanism underlying this phenomenon, as comprehensively described by Chen et al., involves the replacement of larger OH^−^ ions (ionic radius 140 pm) with smaller F^−^ ions (ionic radius 133 pm), which increases the electrostatic attraction between oxygen atoms in phosphate tetrahedra. This enhanced electrostatic interaction produces a compressive effect on the PO_4_^3−^ tetrahedron, leading to shorter P-O bonds and, consequently, higher vibrational frequencies [20,38,39].

The observed shift is consistent with trends reported in the literature, where increasing fluorine content results in progressive upward shifts of the ν_1_ PO_4_^3−^ band. The 3 cm^−1^ shift observed in our FAP/glucan composites therefore suggests a substantial degree of fluoride substitution, corroborating findings from other analytical techniques employed in this study.

Analysis of the ν_3_ PO_4_^3−^ region (1000–1100 cm^−1^) revealed significant spectral simplification in FAP-containing samples (Figure 2c). While HAP/glucan composites exhibited the characteristic seven bands in this region consistent with literature reports, FAP/glucan samples displayed only five distinct bands. This reduction in the number of bands indicates increased crystallographic symmetry resulting from fluoride-induced structural ordering. According to Penel et al., pure fluorapatite should exhibit only four bands in this region (1034, 1042, 1053, 1081 cm^−1^), excluding the band at 1063 cm^−1^. The observation of five bands in our FAP/glucan samples confirms incomplete F^−^/OH^−^ substitution, leading to the formation of fluorohydroxyapatite (FOHAP) rather than pure fluorapatite. This partial substitution is commonly observed in sol–gel synthesis methods and does not significantly compromise the beneficial properties of fluoride-containing apatites [40].

The hydroxyl region (3500–3600 cm^−1^) provided crucial evidence for partial fluoride substitution (Figure 2d). The Raman spectra showed a single band around 3573 cm^−1^ for HAP samples and 3537 cm^−1^ for fluoridated apatite. The 3573 cm^−1^ band corresponds to structural hydroxyl groups located within the channels along the c-axis of HAP crystals, while the 36 cm^−1^ downfield shift to 3537 cm^−1^ indicates a change in the chemical environment of OH^−^ groups due to the presence of neighboring F^−^ ions [20].

Remarkably, for the FAP/glucan composite, this signal at 3537 cm^−1^ becomes invisible, which represents an unexpected finding based on structural data and the previous literature. This absence could result from further structural ordering in the presence of the glucan polymer or from specific interactions at the ceramic–polymer interface. The polymer matrix may promote additional fluoride incorporation or cause changes in the vibrational coupling that render this band undetectable. This phenomenon warrants further investigation using high-resolution solid-state NMR techniques to fully understand the local structural environment.

A notable absence in our spectra is the characteristic Ca-F band at 311 cm^−1^, which has been reported for fluorapatite by Penel et al. This absence further confirms incomplete fluoride substitution and supports the formation of fluorohydroxyapatite rather than pure fluorapatite. The Ca-F band typically appears only when significant fluoride incorporation occurs, leading to the formation of strong covalent Ca-F bonds within the crystal structure [40].

Analysis of peak widths (FWHM) for the most intense band at ~960 cm^−1^ indicates enhanced crystallinity in fluoride-containing samples. Narrower bands in FAP/glucan composites compared to HAP/glucan composites suggest better crystallographic ordering, a characteristic feature of fluorinated apatites. Fluoride ions act as structure-stabilizing agents, preventing thermal decomposition at elevated temperatures and promoting crystal growth during thermal treatment processes [39].

The structural modifications revealed by Raman spectroscopy have direct implications for the biological performance of these composites. The P-O bond shortening and increased crystallinity associated with fluoride incorporation result in enhanced chemical stability and reduced solubility in acidic environments, which are advantageous properties for biomedical applications [16,17,18]. Moreover, the controlled release of fluoride ions from FOHAP structures can stimulate osteoblast proliferation, enhance mineralization processes, and provide antimicrobial effects at the implant site.

### 3.3. Fourier Transform Infrared Spectroscopy (FTIR)

**Figure 3:**
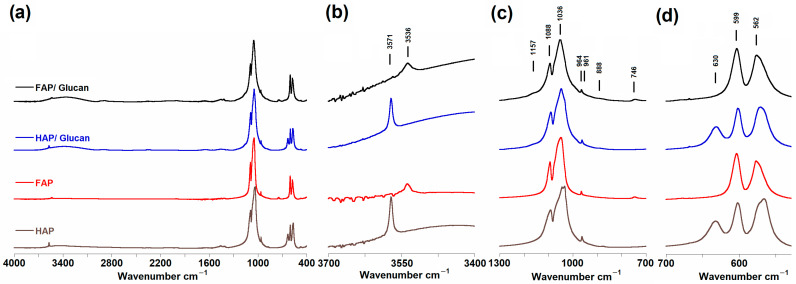
FTIR spectra of HAP, FAP, HAP/glucan, and FAP/glucan samples: (**a**) range 400 to 4000 cm^−1^; (**b**) range 3400 to 3700 cm^−1^ (magnified ×10); (**c**) range 900 to 1200 cm^−1^; (**d**) range 500 to 700 cm^−1^ (magnified ×2).

The FTIR spectra obtained for all samples are presented in Figure 3. The spectroscopic analysis confirmed the preservation of characteristic bands observed in pure HAP and FAP within the obtained curdlan composites. All spectra exhibited a prominent PO_4_^3−^ band near 1036 cm^−1^, along with several bands within the 500–650 cm^−1^ range and a weak band between 3500 and 3600 cm^−1^ associated with OH groups. The presence of β-1,3-glucan in the composite samples was evidenced by characteristic polymer bands at 888 cm^−1^ and 1157 cm^−1^, attributed to C1-O-C3 stretching vibrations specific to the β-configuration of the polysaccharide backbone. These bands serve as definitive markers for glucan incorporation and demonstrate successful composite formation [20,41,42].

As shown in Figure 3b, the FAP and FAP/glucan samples display a characteristic shift in the OH group signal from 3571 cm^−1^ to 3536 cm^−1^. This 35 cm^−1^ downfield shift toward lower wavenumbers indicates a change in the chemical environment of hydroxyl groups due to the presence of neighboring F^−^ ions, consistent with the mechanism described by Freund and Knobel [43]. The observed shift confirms effective fluoride incorporation into the apatite crystal lattice, as fluorine introduction into the hydroxyapatite structure leads to systematic changes in OH^−^ group vibrations. In fluoride-containing samples, the differential signal at 630 cm^−1^ attributed to non-apatitic P-OH groups disappeared, which correlates with a significant reduction in intensity of the OH group signal in the 3571–3536 cm^−1^ range. This effect confirms the effective substitution of hydroxyl groups by fluoride ions in the apatite structure [19,20].

Of particular significance is the emergence of a distinctive band at 746 cm^−1^ in all FAP-containing samples (Figure 3c), which was absent in pure HAP samples. This band has been attributed by Freund and Knobel to OH groups involved in –OH- - - F– hydrogen bonding interactions, where residual hydroxyl groups form specific hydrogen bonds with fluoride ions within the apatite channels [43]. The presence of this band provides direct spectroscopic evidence for partial fluoride substitution, indicating the formation of fluorohydroxyapatite (FOHAP) rather than pure fluorapatite. Wei et al. similarly used the presence of the 746 cm^−1^ band as an indicator of fluoride ion incorporation in fluorapatite structures, while Veiderma et al. observed identical spectral characteristics in fluoride-rich apatite samples [44,45]. The intensity of this band correlates directly with the degree of fluoride substitution and the extent of F^−^/OH^−^ interactions within the crystal structure.

However, Knubovets suggested an alternative interpretation, proposing that this band may also be attributed to symmetric valence oscillations of P-O-P bridge bonds in apatite spectra [46]. This ambiguity highlights the complexity of vibrational assignments in fluoride-substituted apatites and the need for complementary analytical techniques to confirm structural interpretations. In the context of this study, the correlation between the 746 cm^−1^ band appearance and other spectroscopic evidence for fluoride incorporation (including XRD, Raman, and NMR data) strongly supports the assignment to F^−^/OH^−^ interactions rather than P-O-P bridging bonds.

The polymer-specific bands at 888 cm^−1^ and 1157 cm^−1^ exhibited subtle but consistent differences between HAP/glucan and FAP/glucan composites. In HAP/glucan samples, these bands appeared with higher relative intensity and slightly sharper line shapes compared to FAP/glucan composites, where they showed some broadening and reduced intensity. This observation suggests differential interactions between the glucan polymer and the two ceramic phases, potentially related to differences in surface chemistry and hydrogen bonding capacity. The reduced intensity of glucan bands in FAP/glucan composites may indicate stronger ceramic–polymer interactions or changes in the local conformational state of the polysaccharide chains when in contact with the fluoride-modified apatite surface [41].

The phosphate region (900–1200 cm^−1^) showed characteristic modifications associated with fluoride substitution (Figure 3c). The main ν_1_ PO_4_^3−^ band remained relatively stable at ~1036 cm^−1^, however, subtle changes in intensity and band width were observed, which may be related to structural ordering changes induced by the presence of F^−^ ions. Analysis of the ν_4_ PO_4_^3−^ region (500–650 cm^−1^) revealed the presence of characteristic bands at 581, 592, 608, and 617 cm^−1^ in HAP samples, consistent with literature data. In FAP samples, simplification of this region was observed with dominant bands at 608 and 591 cm^−1^, indicating increased crystallographic symmetry induced by fluoride substitution [19,20].

The obtained FTIR spectroscopic results are consistent with observations by Penel and coworkers, who described similar spectral changes in a series of fluorine-substituted apatites. The characteristic shift of the OH band from 3573 cm^−1^ (for pure HAP) to the 3536–3540 cm^−1^ range (for fluorine-containing samples), combined with the appearance of the 746 cm^−1^ band, confirms the formation of fluorohydroxyapatite (FOHAP) rather than pure fluorapatite. The absence of the OH librational band at 630–655 cm^−1^, often described in the literature, may indicate specific synthesis conditions employed in this work or the high efficiency of F^−^/OH^−^ substitution in the obtained materials. The lack of detection of characteristic bands for pure fluorapatite, such as the Ca-F stretching mode around 311 cm^−1^ reported by Penel et al., further supports the formation of fluorohydroxyapatite with partial fluoride substitution [19,20,40,42,43].

The preservation and distinct behavior of the glucan-specific bands at 888 cm^−1^ and 1157 cm^−1^ throughout the composite formation process demonstrates that the polymer maintains its structural integrity while forming intimate contact with both ceramic phases. The subtle differences observed in these bands between HAP/glucan and FAP/glucan composites provide valuable insights into ceramic–polymer interfacial interactions and suggest that fluoride substitution in the ceramic phase influences the local environment and conformational state of the polysaccharide binder.

The structural changes revealed by FTIR spectroscopy have direct implications for the biological properties of these composites, as fluoride substitution leads to increased chemical stability and reduced solubility in acidic environments, which are advantageous properties for biomedical applications [16,17,18]. The spectroscopic evidence for partial F^−^/OH^−^ substitution, particularly the characteristic 746 cm^−1^ band, provides optimal balance between the enhanced chemical stability of fluorapatite and the bioactivity characteristics of hydroxyapatite, potentially offering superior performance for regenerative medicine applications. The successful incorporation of β-1,3-glucan, as evidenced by the preservation of characteristic polymer bands at 888 cm^−1^ and 1157 cm^−1^, ensures that the composite retains the flexibility and handling properties required for clinical applications while delivering the therapeutic benefits of controlled fluoride release.

### 3.4. Solid-State Nuclear Magnetic Resonance Spectroscopy (ssNMR)

**Figure 4:**
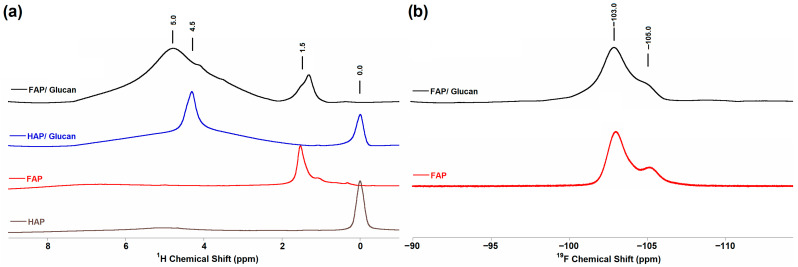
NMR spectra of: (**a**) ^1^H MAS NMR HAP, FAP, HAP/glucan, and FAP/glucan samples; (**b**) ^19^F MAS NMR FAP and FAP/glucan samples.

**Figure 5:**
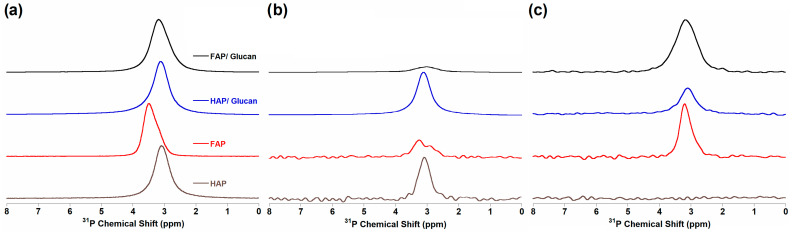
NMR spectra of HAP, FAP, HAP/glucan, and FAP/glucan samples: (**a**) ^31^P BD MAS NMR; (**b**) ^1^H→^31^P CP MAS NMR; and (**c**) ^19^F→^31^P CP MAS NMR.

**Figure 6:**
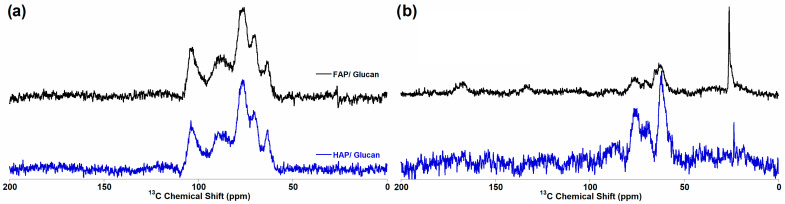
NMR spectra of HAP/glucan and FAP/glucan samples: (**a**) ^1^H→^13^C CP MAS NMR; and (**b**) ^13^C BD MAS NMR.

The solid-state NMR spectra obtained for all investigated samples are presented in Figure 4, Figure 5 and Figure 6. The ^1^H MAS NMR spectra reveal distinct differences between HAP- and FAP-containing materials (Figure 4a). For the HAP sample, two characteristic signals were observed: a sharp signal at 0.0 ppm corresponding to structural OH^−^ groups located within the hexagonal channels along the c-axis, and a broader signal at 5.0 ppm attributed to water molecules adsorbed on the powder surface, consistent with previous literature reports [20,47].

For the FAP sample, the structural OH^−^ signal at 0.0 ppm was absent, confirming effective fluoride substitution. Instead, three distinct signals were observed: a prominent signal at 1.5 ppm, a weaker signal at 1.0 ppm, and a broad signal around 7.0 ppm. The 1.5 ppm signal is characteristic of OH^−^ groups located near F^−^ ions within the channels, arising from the formation of OH^−^-OH^−^-OH^−^-F^−^ motifs instead of the OH^−^-OH^−^-OH^−^-OH^−^ arrangement found in pure hydroxyapatite. This chemical shift provides direct evidence for partial fluoride substitution and the formation of fluorohydroxyapatite (FOHAP) rather than pure fluorapatite. The broad signal at 7.0 ppm corresponds to surface HPO_4_^2−^ species, which exhibit extremely broad line shapes due to structural disorder in the surface layer [20,48].

The composite samples showed additional complexity in their ^1^H NMR signatures. For HAP/glucan, an additional signal appeared around 4.5 ppm, while FAP/glucan exhibited a broader signal around 5.0 ppm. These signals originate from the hydroxyl groups of glucose units in the β-1,3-glucan polymer, specifically from OH groups that form different hydrogen bonding networks when interacting with HAP versus FAP ceramic phases. The different chemical environments of glucan OH groups in the two composites suggest distinct polymer–ceramic interfacial interactions, with the fluoride-modified apatite surface altering the local conformational state of the polysaccharide chains [49].

The ^19^F MAS NMR spectra for fluoride-containing samples (Figure 4b) were recorded at a 20 kHz spinning frequency and displayed characteristic signals in the −103 to −105 ppm range. Based on literature assignments, the signal at −103 ppm corresponds to fluorapatite in crystalline domains, −104 ppm indicates apatite-like boundary layers characteristic of FOHAP, and −105 ppm represents amorphous phases. The presence of multiple fluorine environments confirms the formation of fluorohydroxyapatite with mixed F^−^/OH^−^ occupancy rather than pure fluorapatite, consistent with XRD and spectroscopic findings [20,48].

^31^P MAS NMR analysis employed three complementary techniques: single pulse-acquire (Bloch decay, BD), ^1^H→^31^P cross-polarization, and ^19^F→^31^P cross-polarization (Figure 5). The ^31^P BD experiments (Figure 5a) revealed the main phosphorus signal at approximately 3.1 ppm for HAP samples and 3.5 ppm for fluoride-substituted materials. This 0.4 ppm downfield shift in FAP samples indicates the presence of F^−^ ions in the channels along the c-axis, which alters the electronic environment of phosphorus atoms through structural modifications of the apatite lattice [20,48].

Notably, the FAP/glucan composite showed a signal position intermediate between pure FAP and HAP, suggesting partial fluoride loss or structural reorganization during composite formation. This observation indicates that the glucan gelation process may influence the local fluoride distribution within the apatite structure, possibly through polymer–ceramic interactions that modify the channel environment.

The ^1^H→^31^P cross-polarization experiments (Figure 5b) selectively detected phosphorus nuclei with protons in their vicinity. The signal intensity was significantly reduced for fluoride-containing samples compared to HAP samples, reflecting the lower concentration of OH^−^ groups in fluoride-substituted apatites. This experiment unambiguously confirmed the FOHAP nature of the synthesized fluoride-substituted materials by demonstrating residual OH^−^ group presence.

Conversely, the ^19^F→^31^P cross-polarization experiments (Figure 5c) revealed phosphorus nuclei in fluoride-rich environments. As expected, no signal was observed for HAP samples, while all fluoride-substituted materials displayed characteristic signals around 3.5 ppm. The clear shift toward 3.5 ppm in these experiments confirms the presence of F^−^ ions in the channels along the c-axis and their influence on the phosphorus electronic environment [20].

Carbon-13 NMR analysis confirmed successful glucan incorporation in both composite systems (Figure 6). The ^1^H→^13^C cross-polarization (Figure 6a) and ^13^C Bloch decay (Figure 6b) spectra displayed characteristic glucan signals with carbon assignments consistent with literature reports for β-1,3-glucan. The carbon signals appeared at identical positions in both HAP/glucan and FAP/glucan composites, indicating that the polymer backbone structure remains unchanged despite different ceramic–polymer interfacial interactions evidenced by ^1^H NMR.

Signal assignments for glucan carbons (Table 2) showed C1 at 104 ppm, C2 at 75 ppm, C3 at 88 ppm, C4 at 70 ppm, C5 at 78 ppm, and C6 at 63 ppm, consistent with the expected β-1,3-glucan configuration. The preservation of these characteristic chemical shifts confirms that composite formation does not significantly alter the polysaccharide structure, while the differences observed in ^1^H NMR reflect changes in local hydrogen bonding environments rather than fundamental structural modifications [50,51].

The solid-state NMR analysis provides comprehensive evidence for the successful synthesis of fluorohydroxyapatite-based composites with controlled fluoride substitution levels. The complementary information from ^1^H, ^19^F, and ^31^P NMR techniques confirms partial F^−^/OH^−^ substitution, preservation of the apatite structure, and successful polymer incorporation. The subtle differences in NMR signatures between pure ceramics and composites suggest specific ceramic–polymer interactions that may influence material properties and biological performance, warranting further investigation in cellular studies.

### 3.5. Microscopic Imaging

**Figure 7:**
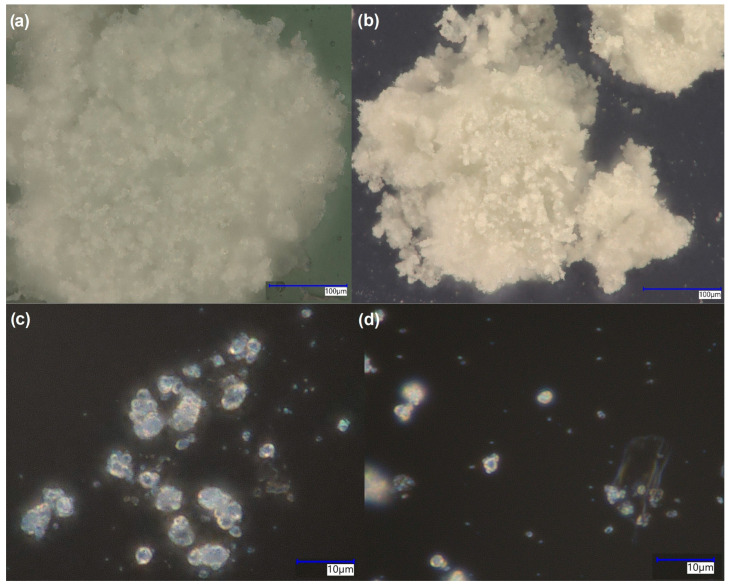
Microscopic imaging: HAP/glucan (panels (**a**,**c**)) and FAP/glucan (panels (**b**,**d**)) composites after grinding in a cryogenic mill. Magnification 20× (panels (**a**,**b**)) and 40× (panels (**c**,**d**)).

The optical microscopy images in Figure 7 reveal distinct morphological differences between the composite systems. At low magnification, both samples display composite agglomerates formed during the mixing and gelation process. However, at higher magnification, two distinct particle populations are evident: particles with sharp, well-defined crystalline edges and particles with diffuse, rounded boundaries that appear ‘amorphous-like’ under optical observation.

This apparent amorphization does not reflect true glass formation but rather results from the intense mechanical processing during composite preparation. The high-energy mixing, sonication, and intimate polymer infiltration during β-1,3-glucan gelation disrupted the surface crystallinity of the original ceramic granules, creating nanoscale disorder at particle interfaces while preserving core crystallinity (as confirmed by XRD). This surface disorder manifests optically as blurred particle boundaries and reduced optical contrast compared to the sharp facets of unprocessed crystalline regions.

Particle size analysis revealed significant differences between the composites: HAP/glucan particles averaged ~2 μm, while FAP/glucan particles were notably smaller at ~1 μm. This size reduction in FAP composites correlates with the enhanced ceramic–polymer interactions evidenced by spectroscopic analysis, suggesting that fluoride substitution promotes more effective polymer infiltration and particle size reduction during composite formation. The smaller particle size in FAP/glucan, despite FAP having larger individual crystallites (confirmed by TEM), indicates more extensive fragmentation during processing, likely due to altered surface chemistry and polymer binding characteristics introduced by fluoride substitution.

### 3.6. Transmission Electron Microscopy (TEM) with Energy-Dispersive X-Ray Spectroscopy (EDS)

**Figure 8:**
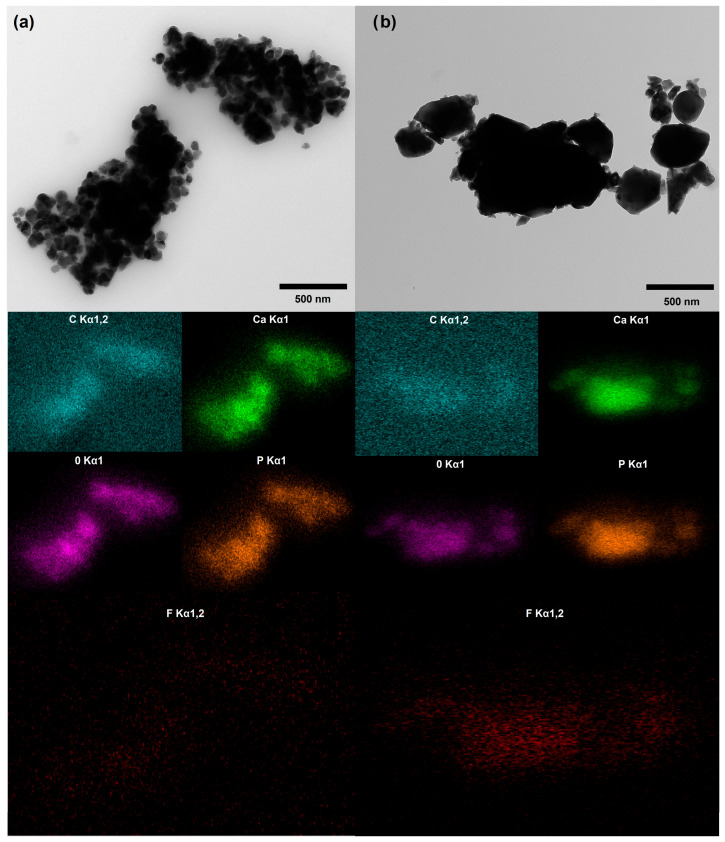
TEM pictures of (**a**) HAP/glucan, (**b**) FAP/glucan. The images below show the elemental distribution maps: C, Ca, P, O, and F obtained by EDS. Fluor EDX analysis photos enlarged 2×.

The TEM images in Figure 8 reveal distinct differences in crystal morphology between the composite systems. Prior to TEM analysis, samples were processed in a cryogenic mill to expose internal crystal structures for proper EDX elemental mapping, which may have influenced the observed crystal dimensions through mechanical fragmentation. Despite this processing effect, clear morphological differences are evident.

Crystals in the HAP/glucan composite appear smaller and more fragmented compared to those in the FAP/glucan composite, which display larger, more intact crystalline domains. This size difference likely reflects the differential response of the two apatite phases to mechanical stress during cryogenic grinding. HAP, with its more open channel structure containing larger OH^−^ ions, may be more susceptible to fragmentation along cleavage planes compared to FAP’s more compact lattice structure resulting from smaller F^−^ ion substitution.

Additionally, the different ceramic–polymer interfacial interactions observed spectroscopically may contribute to varying degrees of crystal embedding within the glucan matrix. HAP crystals, showing stronger polymer adhesion (evidenced by broader glucan bands in FTIR), may experience more effective stress transfer during grinding, resulting in enhanced fragmentation. Conversely, FAP crystals, with their altered surface chemistry due to fluoride substitution, may exhibit different polymer binding characteristics that provide some protection against mechanical damage.

The EDX elemental mapping confirmed uniform distribution of the main composite elements (C, O, Ca, P) throughout both samples, validating successful composite formation. The FAP/glucan sample displayed homogeneous fluorine distribution, confirming complete fluoride incorporation within the apatite lattice, whereas HAP/glucan shows minimal fluorine content below detection limits for meaningful distribution analysis. This elemental homogeneity, combined with the preserved crystal structure differences observed in TEM, demonstrates that the cryogenic grinding successfully exposed internal structures without significantly altering the fundamental compositional characteristics of each composite system.

### 3.7. Internal Microarchitecture

**Figure 9:**
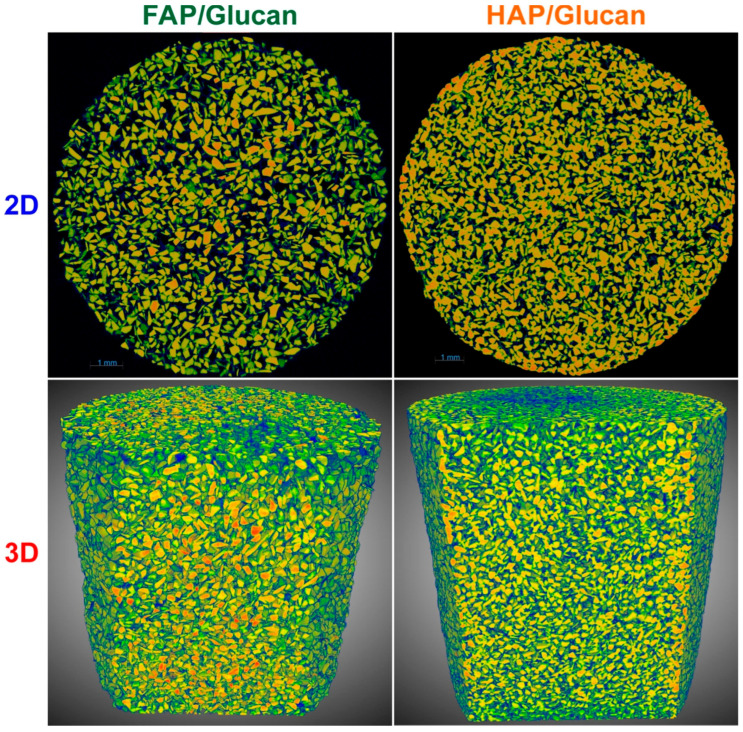
Representative micro-computed tomography (microCT) images of the samples, illustrating the distribution of granules and the intergranular spaces. The upper panels show two-dimensional sections and the lower panels show three-dimensional sections.

The microstructure of the samples was imaged using microCT and was presented in Figure 9. Cross-sectional images illustrate a spatially irregular but overall homogeneous distribution of apatite granules within the sample matrix. The dark (black) regions visible between the apatite granules indicate intergranular voids, which are filled with a polysaccharide phase not visible in the microCT images due to its low radiodensity. The FAP granules have a higher density compared to HAP granules, and therefore occupy a smaller volume in the microCT images. This results in larger apparent spaces between the granules. The percentage distribution of “pores” (space between granules) in the samples is presented in Supplementary Figure S2.

### 3.8. Surface and Porosity

The physical and chemical properties of a material’s surface can influence its behavior and performance in various applications. BET analysis provided valuable insights into the surface area, pore structure, and surface texture of the materials. The results in Table 3 reveal a marked difference in surface area between the two samples: sample FAP/glucan exhibited a surface area of 0.5 m^2^/g (±0.02), while sample HAP/glucan demonstrated a significantly higher surface area of 14.15 m^2^/g (±0.12). The reduced surface area of the FAP/glucan composite, compared to HAP/glucan, can be attributed to the lower pore volume, less developed pore structure, and higher density of the FAP ceramic phase than HAP. The study also showed that the FAP/glucan material had a smaller pore volume than its HAP-containing counterpart; however, the pores were larger (a larger pore width and mesopore diameter).

The reduced pore volume observed in the FAP/glucan composite can be attributed to several interrelated structural, morphological, and physicochemical factors. Fluorapatite (FAP) possesses a smaller unit cell volume (523–524 Å^3^) compared to hydroxyapatite (HAP) (528–529 Å^3^), due to the substitution of larger hydroxyl (OH^−^) ions with smaller fluoride (F^−^) ions [20,52,53,54]. This ion replacement results in a tighter crystal lattice, leading to denser and less porous crystal packing. Such nanoscale densification reduces the accessible meso- and microporosity, which in turn lowers the specific surface area and overall pore volume. Additionally, microCT imaging revealed that FAP/glucan composites exhibit less densely packed granules than HAP/glucan. While this might initially appear contradictory—since less dense packing would normally suggest increased void space—if the intergranular voids are closed or isolated, they may not be accessible to N_2_ gas during adsorption measurements, resulting in an apparently lower pore volume [55,56]. Another possible explanation lies in surface chemistry and hydrophilicity differences. FAP contains fewer hydroxyl groups, which alters surface energy and interactions with glucan. The reduced presence of OH^−^ groups may decrease surface hydrophilicity and affinity for water or gas adsorption, rendering some pores less accessible or inactive during gas adsorption tests [57,58]. This effect could lead to an underestimation of actual porosity or indicate functional pore closure within the polymer matrix. Finally, since glucan is a polysaccharide, it may interact differently with FAP compared to HAP. The altered surface chemistry caused by fluorine substitution could modify the ceramic–polymer interfacial structure, potentially promoting greater polymer infiltration or pore collapse, which effectively reduces the measurable pore volume.

At this stage, it is important to consider how the reduced porosity and specific surface area of the FAP/glucan composite may influence cellular responses. Firstly, a lower specific surface area results in a reduced contact interface for cell adhesion. Cells such as osteoblasts require a sufficiently developed surface to anchor effectively. A decrease in available surface area limits the number of integrin binding sites, which may lead to slower adhesion, reduced cell spreading, and potentially delayed differentiation [59]. Secondly, diminished porosity can restrict the transport of nutrients and metabolic waste. Pores play a crucial role in facilitating the diffusion of oxygen and nutrients, as well as the removal of cellular byproducts. A lower pore volume may compromise the local microenvironment within the implant, negatively affecting cell viability and proliferation [60]. Finally, the reduced porosity and surface area may also impact the resorption behavior of the material. Less porous structures are typically more mechanically stable and degrade at a slower rate, which could be advantageous in long-term biomedical applications where sustained structural support is required [61].

The potential advantages and limitations of FAP/Glucan composites may be partially offset, depending on the specific requirements of a given biomedical application. A key functional role of FAP within such materials is the localized release of fluoride ions, which has been associated with enhanced osteogenic cell proliferation, stimulation of osteoblast differentiation, and accelerated mineralization processes [62,63,64].

Surface roughness measurements obtained using an optical profilometer are presented in Table 4, while 3D images of the surface topography are shown in Figure S3 (Supplementary Materials). The average roughness (Ra) was 34.1 µm for the HAP/glucan composite and 27.6 µm for the FAP/glucan composite; however, this difference was not statistically significant (*p* = 6.49%). Similarly, the root mean square roughness (Rq) values showed no statistically significant difference between the materials, with values of 42.8 µm for HAP/glucan and 37.6 µm for FAP/glucan (the variation in Rq for the FAP composite was relatively large, which contributed to the lack of statistical significance).

Although the average surface roughness (Ra) and root mean square roughness (Rq) values for both HAP/glucan and FAP/glucan composites were not statistically significantly different, the slightly lower roughness observed in the FAP/glucan composite may still influence cellular responses. Surface roughness at the microscale can significantly affect cell adhesion, proliferation, and differentiation, particularly for osteoblasts and other bone-related cells [59,60]. Moderate surface roughness has been shown to enhance osteoblastic activity and promote better cell attachment and spreading, thereby facilitating osteointegration and tissue regeneration [65,66]. Since both composites exhibit comparable roughness values within a similar range (~27–34 µm Ra), their ability to support initial cell adhesion and subsequent biological activity is expected to be similar. However, the somewhat higher variability in roughness in the FAP composite may lead to localized heterogeneity in cell responses [67]. In summary, the comparable surface roughness suggests that both composites provide a suitable microenvironment for cell attachment and growth. Nonetheless, slight differences in roughness distribution, alongside other physicochemical properties, may modulate biological performance. These aspects warrant further investigation through in vitro cell culture studies to evaluate the effects on cell viability, proliferation, and differentiation [68].

The observations presented herein highlight the complex relationship between the type of apatite used and the resulting physical and chemical properties of the composite specimens. It is important to emphasize that the observed changes apply specifically to the studied composite materials—namely, β-1,3-glucan polysaccharide combined with hydroxyapatite and/or fluorapatite granules sintered at 800 °C. As such, the absolute values obtained are of limited standalone significance. However, the underlying mechanisms driving these changes, depending on the specific apatite used, are broadly applicable. They illustrate the general trends in property variations across entire samples and scaffolds, particularly those intended for applications in regenerative medicine, where interaction with living cells is expected. The importance of such investigations has been widely acknowledged in the literature [69,70,71,72,73], reflecting the increasing demand for advanced biomaterials in this field. A thorough understanding of the mechanism of chemical and structural changes occurring in composite materials depending on the type of apatite used will allow us to obtain a product with the desired parameters. Further research using cell lines will clarify how these physicochemical changes translate into biological responses.

A comparative analysis between our ceramic–polymer composites (β-1,3-glucan combined with hydroxyapatite or fluorapatite granules sintered at 800 °C) and existing materials for bone tissue regeneration reveals several distinctions in terms of composition, fabrication methods, and resulting properties. While existing materials for bone tissue regeneration focus on enhancing mechanical properties and bioactivity, our composites introduce a novel combination of β-1,3-glucan with calcium phosphates, offering unique structural and biological properties. The choice of fabrication techniques and material combinations significantly influences the resulting properties and potential applications in regenerative medicine [74].

## 4. Conclusions

Replacing calcium phosphate granules (HAP) with fluoride-substituted calcium phosphate granules (FAP) significantly altered the physicochemical properties of the ceramic–polymer composites. Based on comprehensive chemical, structural, and surface analyses, the following conclusions can be drawn:

1. FAP-based composites were denser and less porous, with significantly smaller agglomerates (~1 µm) and markedly lower specific surface area (0.50 m^2^/g) compared to HAP/glucan composites (~2 µm; 14.15 m^2^/g), contributing to enhanced mechanical stability.
2. The F^−^/OH^−^ ion exchange in FAP resulted in a contraction of the unit cell volume (524.6 Å^3^ vs. 528.2 Å^3^ for HAP), accompanied by characteristic spectral changes such as a shift in the OH stretching band (3573 → 3537 cm^−1^) and the appearance of a new band at 746 cm^−1^, which supports sustained fluoride release.
3. HAP-based composites retained higher porosity (pore volume 0.03 cm^3^/g vs. 0.002 cm^3^/g) and larger particle sizes, favoring greater fluid uptake and potentially enhanced osteoconductive properties.
4. Surface topographical analysis revealed that FAP/glucan composites exhibited sharper profile peaks (Rp 352 µm vs. 134 µm) despite similar average roughness values (Ra ~ 30 µm), suggesting tailored tissue–material interactions through modified surface features.
5. Microstructural observations confirmed a homogeneous distribution of ceramic granules in both composites; however, FAP/glucan composites showed a less densely packed granule arrangement than HAP/glucan.
6. Microscopic and elemental analyses indicated that FAP/glucan composite particles were smaller (~1 µm) but composed of larger individual crystals compared to HAP/glucan (~2 µm), with uniform distribution of major elements (C, O, Ca, P) and distinct fluorine localization.
7. Spectroscopic techniques (Raman, FTIR, and NMR) further revealed distinct chemical signatures in FAP composites, including upfield shifts of phosphate bands, shifts in OH group vibrations, and changes in ^31^P NMR signals indicative of P–O bond shortening due to fluoride incorporation.
8. Polymer–ceramic interfacial interactions differed between composites: glucan-specific bands (888 and 1157 cm^−1^) were sharper in HAP composites and broadened in FAP composites, which may suggest stronger binding interactions in the fluoride-substituted material.
9. Despite attempts for complete fluoride substitution, residual hydroxyl groups persisted in FAP samples, particularly at crystal edges and surfaces, indicating incomplete F^−^/OH^−^ exchange.

## Figures and Tables

**Table 1:** Crystal parameters of granules and composites determined from PXRD analysis.

Sample	a (Å)	c (Å)	Cell Volume (Å^3^)	Crystallite Size Along c-Axis	Crystallite Size Along a-Axis
HAP	9.415(2)	6.881(1)	528.2(1)	30 ± 2 nm	24 ± 2 nm
FAP	9.379(3)	6.885(2)	524.6(3)	58 ± 4 nm	49 ± 4 nm
HAP/Glucan	9.419(3)	6.881(2)	528.7(3)	34 ± 2 nm	31 ± 2 nm
FAP/Glucan	9.381(2)	6.888(2)	525.0(3)	51 ± 3 nm	47 ± 3 nm

**Table 2:** Signal positions of glucan carbons in the ^1^H→^13^C CP MAS NMR spectrum [50,51].

	C1	C2	C3	C4	C5	C6
HAP/Glucan	104 ppm	75 ppm	88 ppm	70 ppm	78 ppm	63 ppm
FAP/Glucan	103 ppm	75 ppm	88 ppm	70 ppm	78 ppm	63 ppm

**Table 3:** Surface area and porosity values determined by low-temperature nitrogen adsorption (reported as mean ± standard deviation).

Parameter		Sample		Statistically Significant Difference *
	Units	HAP/Glucan Mean (±SD)	FAP/Glucan Mean (±SD)	
S_BET_	[m^2^/g]	14.15 (±0.12)	0.5 (±0.02)	yes, *p ≤ 0.0001*
S_micro_	[m^2^/g]	1.82 (±0.46)	0.04 (±0.06)	yes, *p = 0.0026*
S_ext_	[m^2^/g]	12.32 (±0.38)	0.47 (±0.06)	yes, *p ≤ 0.0001*
mesopore diameter	[nm]	9.54 (±0.21)	15.75 (±0.59)	yes, *p ≤ 0.0001*
maximum pore volume	[cm^3^/g]	0.03 (±0)	0.002 (±0)	yes, *p ≤ 0.0001*
median pore width	[nm]	36.04 (±0.62)	82.84 (±5.06)	yes, *p ≤ 0.0001*

* Statistically significant differences between composites according to unpaired Student’s *t*-test.

**Table 4:** Material roughness parameters obtained using an optical profilometer (mean, ±SD).

Parameter	Sample		Statistically Significant Difference *
	HAP/Glucan Mean (±SD)	FAP/Glucan Mean (±SD)	
Ra (roughness average)	34.1 (±3.49)	27.6 (±9.868)	no, *p = 0.0649*
Rq (root mean square average)	42.8 (±3.665)	37.6 (±10.98)	no, *p = 0.1781*
Rp (maximum profile peak height)	134 (±18.96)	352 (±54.12)	yes, *p ≤ 0.0001*
Rv (maximum profile valley depth)	−231.6 (±34.61)	−273.3 (±44.66)	yes, *p = 0.0315*
Rt (maximum height of the profile)	365.7 (±42.25)	625.3 (±77.2)	yes, *p ≤ 0.0001*

* Statistically significant differences between composites according to unpaired Student’s *t*-test.

## Data Availability

The original contributions presented in this study are included in the article/Supplementary Material. Further inquiries can be directed to the corresponding authors.

## Supplementary Materials

The following supporting information can be downloaded at: https://www.mdpi.com/article/10.3390/ma18194538/s1, Figure S1. Diagram showing basic surface roughness parameters (based on X). The green circles indicate the most important parameters. The letter ‘L’ is the length over which the values of surface parameters are evaluated and the letter ‘M’ is the mean line (the reference line about which the profile deviations are measured). Figure S2. Graphs showing the percentage distribution of pores (space between granules). Data are from microCT. Figure S3. Representative 3D images of the surface topography and morphology of the FAP/glucan (top) and HAP/glucan (bottom) composites are shown for a scanned area of 1750 µm × 2333 µm. Roughness parameters were averaged over the entire analyzed surface. The color scale adjacent to each image indicates variations in scanning depth, with blue representing regions below the reference plane and red indicating areas above it.

## Abbreviations

The following abbreviations are used in this manuscript:

FAP	fluorapatite
FAP/Glucan	fluorapatite/β-1,3-glucan composite material
FTIR	Fourier transform infrared spectroscopy
HAP	hydroxyapatite
HAP/Glucan	hydroxyapatite/β-1,3-glucan composite material
microCT	micro-computed tomography
NMR	nuclear magnetic resonance
PXRD	powder X-ray diffractometry
TEM	transmission electron microscopy
