# Supplementary material for: Alterations in the Physicochemical and Structural Properties of a Ceramic–Polymer Composite Induced by the Substitution of Hydroxyapatite with Fluorapatite
Source: Materials (Basel). 2025 Sep 29;18(19):4538. doi: 10.3390/ma18194538 (PMC12525608; doi:10.3390/ma18194538)
Supplement: Supplementary file 1 [file materials-18-04538-s001.zip › materials-3871807-supplementary.pdf]

materials

Article

Alterations in the Physicochemical and Structural Properties of a Ceramic–Polymer Composite Induced by the Substitution of Hydroxyapatite with Fluorapatite

Leszek Borkowski [1,*], Krzysztof Palka [2] and Lukasz Pajchel [3,*]

1 Chair and Department of Biochemistry and Biotechnology, Medical University of Lublin, Chodzki 1, 20-093 Lublin, Poland
2 Faculty of Mechanical Engineering, Lublin University of Technology, Nadbystrzycka 36, 20-618 Lublin, Poland; k.palka@pollub.pl
3 Chair of Analytical Chemistry and Biomaterials, Department of Analytical Chemistry, Medical University of Warsaw, ul. Banacha 1, 02-097 Warsaw, Poland
* Correspondence: leszek.borkowski@umlub.pl (L.B.); lukasz.pajchel@wum.edu.pl (L.P.)

Supplementary Materials

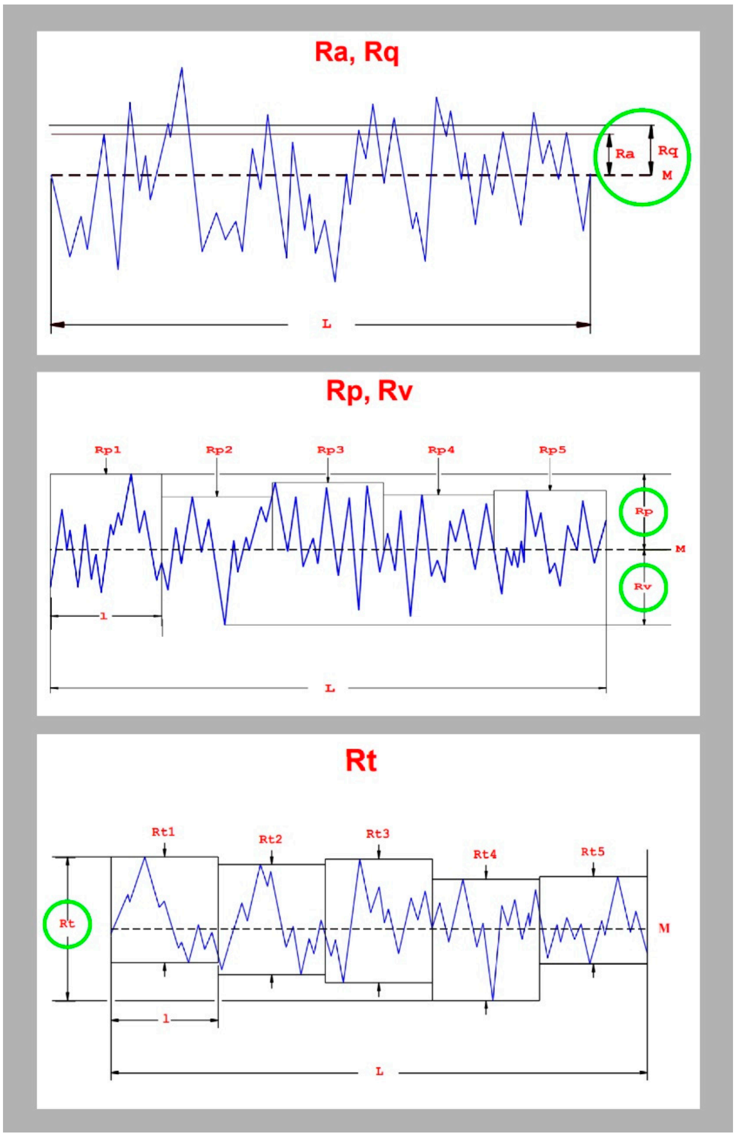

Figure S1. Diagram showing basic surface roughness parameters (based on X). The green circles indicate the most important parameters. The letter 'L' is the length over which the values of surface parameters are evaluated and the letter 'M' is the mean line (the reference line about which the profile deviations are measured).

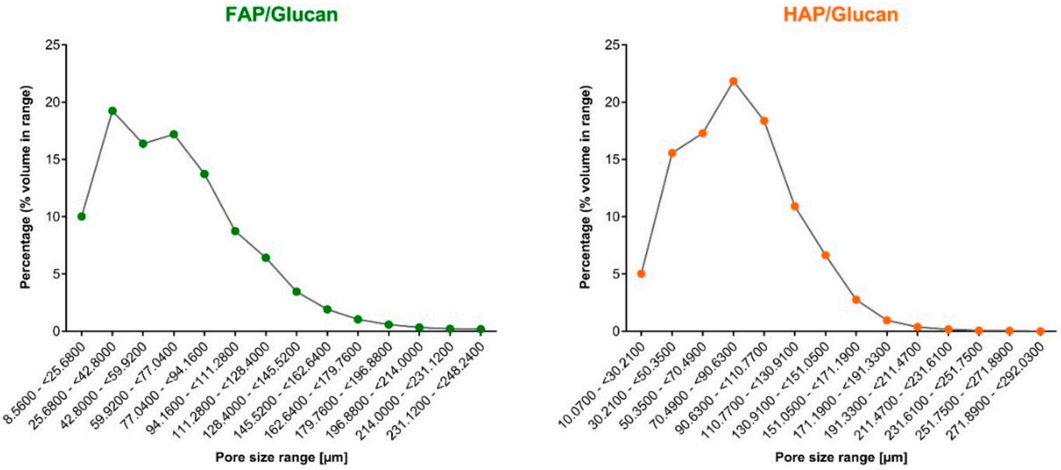

Figure S2. Graphs showing the percentage distribution of pores (space between granules). Data are from microCT.

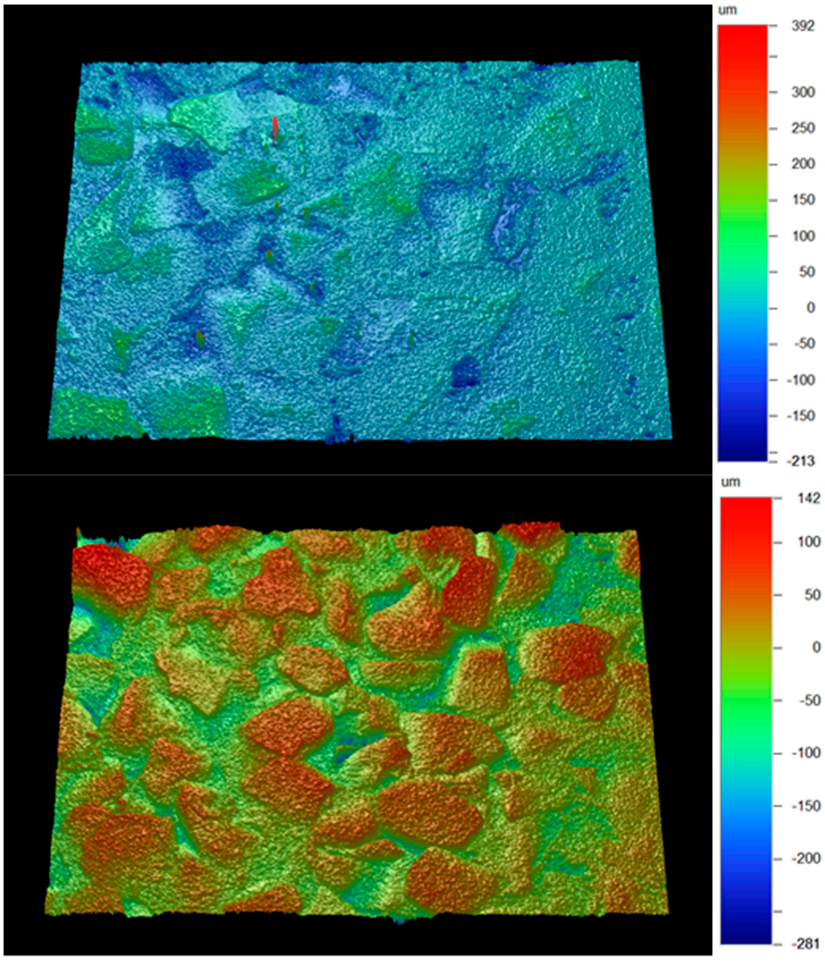

Figure S3. Representative 3D images of the surface topography and morphology of the FAP/Glucan (top) and HAP/Glucan (bottom) composites are shown for a scanned area of 1750 μm × 2333 μm. Roughness parameters were averaged over the entire analyzed surface. The color scale adjacent to each image indicates variations in scanning depth, with blue representing regions below the reference plane and red indicating areas above it.
